# Optimizing Arterial Vessel Contrast in Portal Venous Phase with Virtual Monoenergetic Images from Photon-Counting Detector CT Scans of the Abdomen—First Clinical Experiences

**DOI:** 10.3390/diagnostics14060627

**Published:** 2024-03-15

**Authors:** Daniel Dillinger, Daniel Overhoff, Isabelle Ayx, Hanns L. Kaatsch, Achim Hagen, Stefan O. Schönberg, Stephan Waldeck

**Affiliations:** 1 Department of Vascular Surgery and Endovascular Surgery, Bundeswehr Central Hospital, Rübenacher Straße 170, 56072 Koblenz, Germany; 2 Department of Radiology and Neuroradiology, Bundeswehr Central Hospital, Rübenacher Straße 170, 56072 Koblenz, Germany; 3 Department of Radiology and Nuclear Medicine, University Medical Centre Mannheim, Medical Faculty Mannheim, Heidelberg University, Theodor-Kutzer-Ufer 1-3, 68167 Mannheim, Germany; 4 Department of Neuroradiology, University Medical Center Mainz, Langenbeckstraße 1, 55131 Mainz, Germany

**Keywords:** computed tomography, computed tomography angiography, virtual monoenergetic imaging, contrast noise ratio, signal noise ratio, spectral imaging, photon-counting detector CT

## Abstract

Background: Photon-counting detector (PCD) computed tomography (CT) allows for the reconstruction of virtual monoenergetic images (VMI) at different thresholds. Objective: The aim of our study was to evaluate the optimal arterial contrast in portal venous (pv) scans regarding objective parameters and subjective image quality for different virtual keV levels. Methods: We identified 40 patients that underwent a CT scan with an arterial and pv phase on a PCD-CT (NAEOTOM alpha, Siemens Healthineers, Forchheim, Germany). The attenuation of abdominal arteries on pv phases was measured for different virtual keV levels in a monoenergetic+ application profile and for polychromatic (pc) arterial images. Two independent readers assessed subjective image quality, including vascular contrast in pv scans at different energy levels. Additionally, signal- and contrast-to-noise ratios (SNR and CNR) were measured. Results: Our results showed increasing arterial attenuation levels with decreasing energy levels in virtual monoenergetic imaging on pv scans with the highest attenuation at 40 keV, significantly higher than in the pc arterial phase (439 ± 97 HU vs. 360 ± 97, *p* < 0.001). Noise, SNR, and CNR were worse at this energy level (*p* < 0.001). Pv VMI showed less noise at energy levels above 70 keV (all *p* < 0.001). Subjective image quality was rated best at 70 keV, vascular contrast was best at 40 keV. Conclusions: Our research suggests that virtual monoenergetic images at 40 keV in Mono+ mode derived from a PCD-CT can be a feasible alternative to a true arterial phase for assessment of vessels with worse CNR and SNR.

## 1. Introduction

Virtual monoenergetic imaging (VMI) was introduced with dual-energy computed tomography (CT) detector technology [1,2,3,4,5,6] using different acquisition approaches (dual-spin, dual-source, rapid kV switching, split beam) [7,8]. Lately, another technology allowing VMI has become clinically usable—the photon-counting detector (PCD) technique. This technology uses a cadmium telluride detector and allows the deposited energy of the photon to be measured directly, rather than using a scintillator to convert it into a light signal and then detecting the light—requiring two steps to obtain the information. Also, spectral information can be acquired, which allows the image reconstruction at different keV levels.

When virtually lowering keV values, the attenuation of iodine contrast increases, which has already led to a lower amount of contrast media needed for sufficient contrast in PCD-CT [9,10]. In addition, the extermination of the electronic noise in PCD-CT improves the overall image quality [11,12,13,14,15,16,17,18], and PCD scans showed similar or even lower radiation doses compared to energy integrating detector CT scans [12,19].

CT angiography (CTA) is a standard procedure for assessing vascular structures, e.g., prior to endovascular or open operative procedures [20,21,22].

The assessment and quantification of the stenosis of iliac arteries demonstrates a robust association with invasive angiography, thereby establishing its utility as an essential component in pre-interventional planning within clinical practice [23].

Additionally, CTA of the abdomen is the standard of reference for pre-interventional planning of complex vascular procedures such as endovascular aortic repair (EVAR) or fenestrated EVAR (FEVAR) [24].

In non-primary arterial contrast medium phases, incidental findings such as aortic aneurysms or stenoses may, under certain conditions, lack adequate quantification or exhibit reduced sensitivity and specificity in terms of detection. In consideration of the aforementioned benefits of PCD-CT, our research aimed to assess the feasibility of evaluating abdominal arteries in portal venous (pv) phase examinations using improved iodine contrast at lower VMI levels, particularly in scenarios where the contrast phase for arterial vessel evaluation is suboptimal.

## 2. Materials and Methods

This research was approved by the local ethics committee and was conducted in accordance with the Declaration of Helsinki. Written consent was waived due to the retrospective design of this study.

### 2.1. Study Population

**Figure 1:**
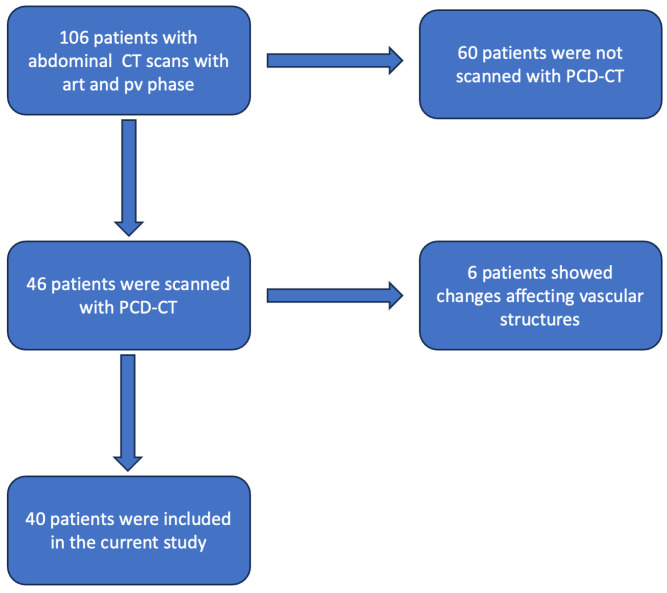
Flowchart demonstrating the patient selection process (art = arterial; pv = portalvenous; CT = computed tomography; PCD-CT = photon counting detector CT).

We identified 106 patients undergoing clinically indicated contrast-enhanced CT scans of the abdomen, including arterial and pv phases, between February 2022 and May 2023. Only patients who had been scanned with the first generation PCD-CT (NAEOTOM alpha Siemens Healthineers, Forchheim, Germany) were included; this left a total of 46 patients, of which 6 had anomalies of the visceral arteries (resections after surgery, metal artifacts resulting from, e.g., coils, affecting vascular structures) and were therefore also excluded. A total of 40 patients were therefore enrolled in the current research (see Figure 1).

### 2.2. Imaging Protocol

All patients were scanned by modulating the tube current in the *z*-axis in accordance with the patient’s diameter and attenuation from the acquired tomograms after the injection of 100 mL contrast medium (Xenetix 350, containing 76.78 g/100 mL Iobitridol, Guerbet, Roissy, France) followed by a saline chaser of 50 mL through an antecubital i.v. catheter with a power injector (CT Motion XD8000, Ulrich Medical, Ulm, Germany).

The data were acquired at 120 kVP. All scans were performed in a cranio-caudal direction with a pitch of 0.80 and an increment of 0.70 mm. The arterial phase scan was started with bolus tracking and a predefined threshold of 140 HU (CARE Bolus software, version VA50A, Siemens Healthineers, Forchheim, Germany) with a delay of 7 s; the pv phase was started at 55 s.

Spectral post-processing (SPP) datasets were used for image reconstruction with a slice thickness of 1 mm with a Qr40 kernel in pv phase. The polychromatic (pc) arterial datasets were reconstructed with a Qr40 kernel as well a slice thickness of 1 mm.

We also retrieved the dose length product (DLP) and CT dose index (CTDI) and measured the patient diameter according to O’Neil et al., which correlates directly with the body mass index (BMI) [25].

### 2.3. Objective Image Parameters

We analyzed the following vessels with dedicated software (Syngio.via, version VB60A_HF04, Siemens Healthineers, Forchheim, Germany) by using the Monoenergetic+ application profile: descending aorta, coeliac trunk, superior mesentery artery, left and right renal artery, as well as left and right common iliac artery. Three regions of interest (ROIs) were placed in equivalent positions in the pv phase (VMI) as well as on arterial pc images of each of the vessels by a radiologist with twelve years of experience.

The ROIs were placed at the energy level of 70 keV, according to Rassouli et al. [26] and Sudarski et al. [6]. We measured the vascular attenuation as well as the standard deviation at the energy levels from 40 keV to 190 keV with increments of 10 keV in VMI in the portal venous phase as well as in arterial pc images.

Additionally, we measured the attenuation and standard deviation in an ROI in the left psoas muscle and extra-abdominal air to calculate the signal-to-noise ratio (SNR) and contrast-to-noise-ratio (CNR) for all energy levels in VMI in the pv phase and again in the arterial pc images.

It was ensured that the ROIs were made as large as possible and that relevant attenuation changes were avoided (e.g., fatty streaks, calcifications).

We calculated CNR and SNR according to Szucs-Farkas et al. [6,27], with

SNR = HUartery/noiseartery

and

CNR = (HUartery − HUmuscle)/noiseair

(noise being the standard deviation in the corresponding measurement).

### 2.4. Subjective Image Analysis

Subjective image quality was assessed by two independent readers (eleven years and twelve years of experience in abdominal radiology).

The readers rated overall image quality, arterial vessel contrast, and image noise on a five-point-Likert scale:

- Image quality:
   5 = optimal;
   4 = good;
   3 = acceptable;
   2 = poor;
   1 = very poor.
- Image noise:
   5 = none;
   4 = minor;
   3 = average;
   2 = more than average;
   1 = major.
- Vessel contrast:
   5 = optimal;
   4 = good;
   3 = acceptable;
   2 = poor;
   1 = not diagnostic.

**Figure 2:**
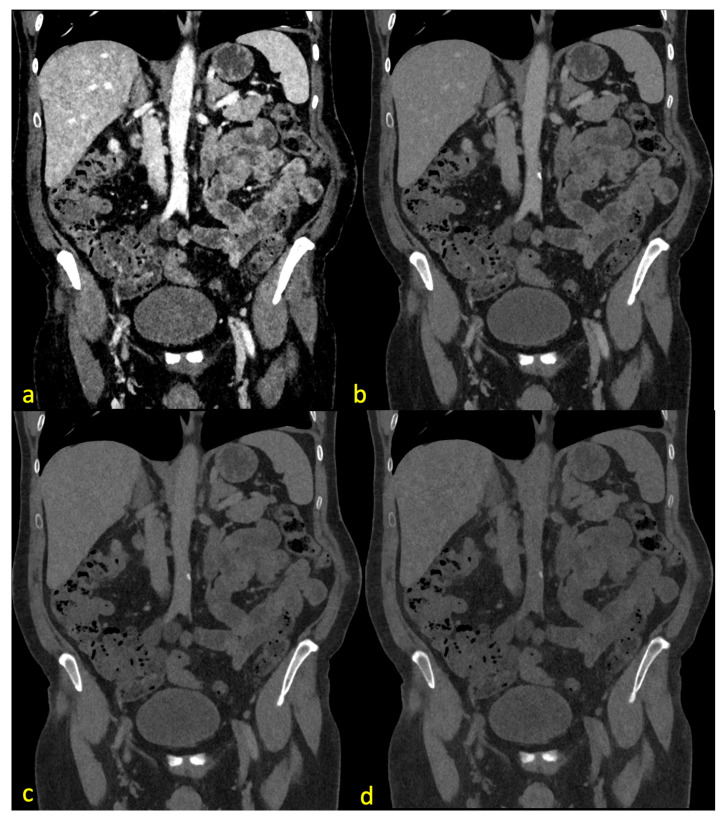
The same CT slice with pv contrast (center 130, width 550) at 40 keV (**a**), 70 keV (**b**), 100 keV (**c**), and 190 keV (**d**). (**a**) demonstrates major image noise with acceptable image quality and optimal vessel contrast. (**b**) shows no noise with a good vessel assessability and optimal image quality. (**c**) shows no noise, acceptable quality, and poor vascular contrast. (**d**) demonstrates no noise, no vessel contrast, and poor image quality.

Figure 2 demonstrates the effects of different energy levels on the CT images, including examples for the different ratings of the Likert scale.

### 2.5. Statistical Analysis

The statistical analysis was performed with SPSS (version 27, IBM Corporation, Armonk, NY, USA). We evaluated categorical and ordinal variables as absolute numbers including their percentages. Interval variables were reported as means and standard deviations. The Shapiro–Wilk-test was applied to test for normal distribution, and a *t*-test for paired samples was performed if data showed a normal distribution; if not, the Mann–Whitney-U-test was performed. A *p*-value < 0.05 was considered statistically significant. To measure the interobserver reliability, we used Cohens Kappa. According to Landis et al. [28], we considered a Kappa of 0 as poor agreement, between 0.00 and 0.20 as slight agreement, 0.21 to 0.4 as fair agreement, 0.41–0.6 as a moderate agreement, 0.61–0.8 as a substantial agreement, and 0.81–1.00 as almost perfect.

## 3. Results

This research included 14 (35%) female patients and 26 males (65%). Further details are shown in Table 1.

### 3.1. Overall Data

The results (means and standard deviation) for vessel density, vessel noise, CNR, and SNR can be seen in Table 2.

#### 3.1.1. Attenuation

**Figure 3:**
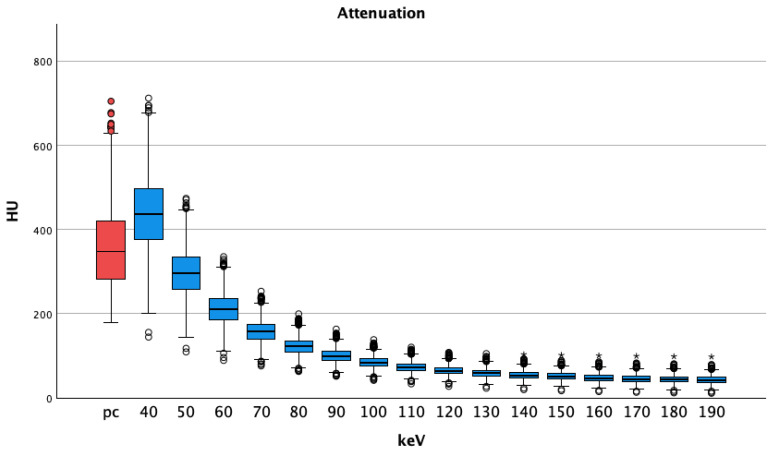
Portal venous phase shows decreasing medians and interquartile ranges of attenuations regarding vessel contrast with increasing energy thresholds. Pv shows higher HU values at 40 keV than pc images; HU = Hounsfield Units pv = portalvenous; pc = polychromatic (arterial), dots show aberrant values, asterisks are more aberrant values.

In pv VMI, only the energy level of 40 keV showed significantly higher attenuation than the regular arterial pc images (VMI pv 40 keV 439 ± 97 HU vs. pc 360 ± 97 *p* < 0.001), increasing the energy level to 50 keV and above. Lower HU values were seen compared to those of pc arterial images (VMI pv 50 keV 298 ± 63 and lower vs. pc 360 ± 97, all *p* < 0.001). Overall, attenuation steadily decreased with increasing energy levels, see Figure 3.

#### 3.1.2. Noise

**Figure 4:**
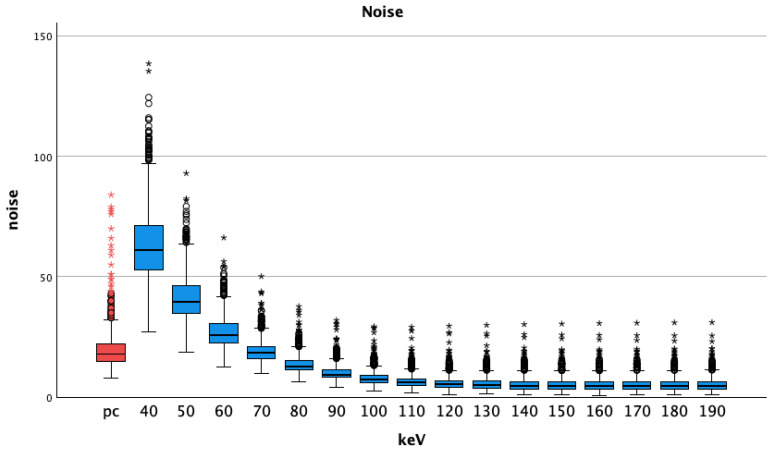
Median and interquartile ranges of noise (standard deviation of the vessel attenuation) shows increasing values with higher energy levels; pc = polychromatic (arterial), dots show aberrant values, asterisks are more aberrant values.

Compared to pc images, the noise in pv VMI showed lower values at the energy levels of 70 keV and above (Table 2). All *t*-tests showed statistically significant differences (*p* < 0.001 for all comparisons, lowest *p* was seen at VMI pv 70 keV 19.22 ± 4.81 vs. pc 19.95 ± 8.58, *p* = 0.022). Noise was highest at 40 keV, showing a steady decrease with increasing energy levels, see Figure 4.

#### 3.1.3. CNR

**Figure 5:**
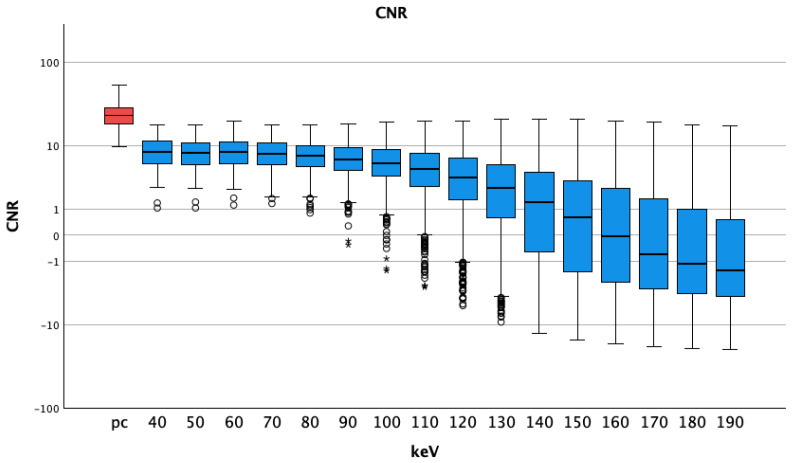
Median and interquartile ranges of contrast-to-noise ratio; they were highest for pc arterial phase, stable at lower energy levels and decreasing for higher thresholds (also with bigger interquartile ranges). A logarithmic scale was used for better visualization; pc = polychromatic (arterial); CNR = contrast-to-noise ratio; dots show aberrant values, asterisks are more aberrant values.

The CNR of arterial pc images (25.10 ± 9.38) was higher than any CNR of VMI pv, (Table 2). The *t*-tests showed *p*-values of less than 0.001 for all comparisons. The CNR reached negative values at energy levels of 170 keV and above (−0.66 ± 4.60). The best CNR for pv VMI was seen at 60 keV (8.64 ± 3.58), see Figure 5.

#### 3.1.4. SNR

**Figure 6:**
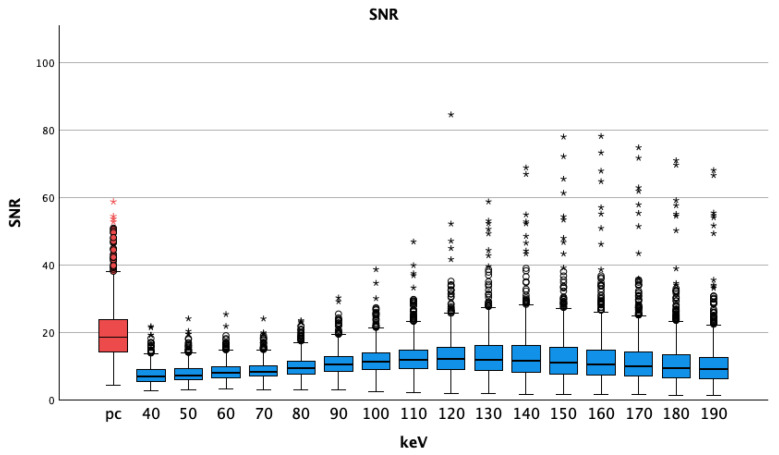
Median and interquartile ranges of signal-to-noise-ratio; pc arterial phase shows higher values compared to the virtual monoenergetic images; SNR = signal-to-noise-ratio; pc = polychromatic (arterial), dots show aberrant values, asterisks are more aberrant values.

The SNR of arterial pc images (20.10 ± 8.30) was significantly higher than that of all VMI of the pv phase at all energy thresholds (Table 2). *P*-values were less than 0.001 for all comparisons of VMI pv vs. pc arterial images. SNR values were highest at 130 keV (13.19 ± 6.88) and lowest at 40 keV (7.42 ± 2.73), see Figure 6.

### 3.2. Vessel Specific Analysis

Additionally, we compared arterial pc images and pv VMI grouped by the examined vessels.

#### 3.2.1. Attenuation

All vessels showed significant differences regarding attenuation with pv VMI at 40 keV, having higher attenuation than arterial pc images and a decrease, which was also seen in the overall data. Means, standard deviations, and *p*-values of the *t*-tests of the vessels at 40 keV are shown in Table 3.

#### 3.2.2. Noise

All pv VMI at 70 keV and above showed lower values for noise than the arterial pc images, besides the right iliac artery (VMI 70 keV 18.37 ± 3.63 vs. pc 17.88 ± 4.07, *p* = 0.358). The comparison of VMI 70 keV with arterial pc images showed no statistical significance for the coeliac trunk (VMI 70 keV 19.12 ± 3.75 vs. pc 19.57 ± 4.077.31, *p* = 0.528), superior mesenteric artery (VMI 70 keV 20.52 ± 4.85 vs. pc 21.25 ± 9.52, *p* = 0.457), right iliac artery (as written above), and right (VMI 70 keV 20.55 ± 6.41 vs. pc 22.59 ± 12.98, *p* = 0.103) and left renal arteries (VMI 70 keV 21.09 ± 6.54 vs. pc 21.46 ± 11.64, *p* = 0.755). All other *t*-tests comparing vessel noise pc vs. VMI at different energy levels showed statistically significant differences with *p*-values less than 0.001.

#### 3.2.3. CNR

The CNR had significant differences in all compared levels, with arterial pc images showing higher CNRs than the pv VMI. The highest CNR of VMI was found for 60 and 70 keV VMIs as in the overall data for all vessels (60 keV).

#### 3.2.4. SNR

The SNR was higher in the arterial pc images in all comparisons. The SNR was lowest at the energy level of 40 keV and showed the highest values in pv VMI for the aorta and right renal artery at 120 keV, coeliac trunk, superior mesenteric artery, right and left iliac arteries at 130 keV, and the left renal artery at 140 keV. All comparisons of VMI pv vs. pc showed statistically significant differences with *p* < 0.001 at all energy levels.

### 3.3. Subjective Image Quality

Cohen’s Kappa for inter-reader agreement regarding subjective image quality showed the following results:

  Substantial agreement for vessel contrast (0.799);
  Substantial agreement for image quality (0.742);
  Substantial agreement for image noise (0.712).

**Figure 7:**
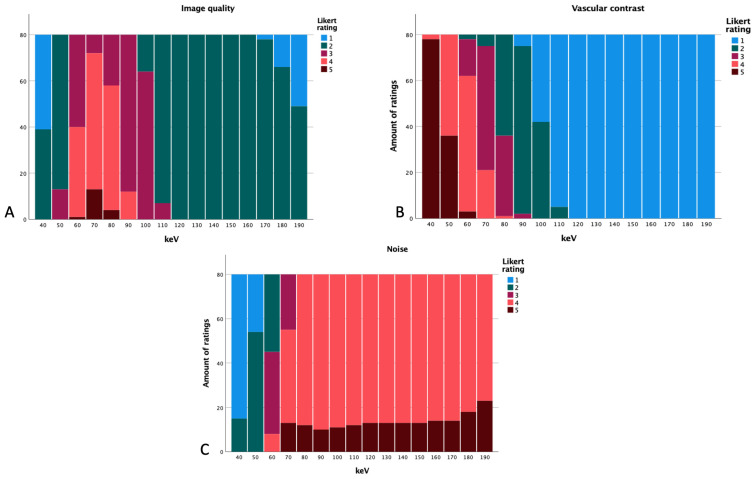
Likert ratings based on the energy level regarding image quality (**A**), vascular contrast (**B**), and subjective noise (**C**).

Th subjective image quality showed the following results:

The best subjective arterial vessel contrast was seen at 40 keV. The noise and overall image quality were rated best at 70 keV (Figure 7). Overall image quality and noise showed ratings of 1 and 2 at 40 keV and most of the ratings for the levels of 110–190 keV for image quality were 2 as well. Image noise showed ratings of 4 and 5 for energy levels of 80 keV and above. Vessel contrast was rated 1 at levels of 110 keV and higher; 40 keV mostly showed ratings of 5 for vascular contrast. We also summed up the scores of the different Likert ratings.

At 40 keV, arterial vessel contrast had a total of 398, noise a total of 95, and overall image quality had 119. At 50 keV, a total of 356 for vessel contrast, 173 for image quality, and 134 for noise were received. By further increasing the energy level to 60 keV, noise showed a total of 213, overall image quality 281,and vessel contrast 303. At the energy level of 70 keV, image quality was rated with a total of 325, vascular contrast with 256, and noise with 308. Noise was rated with more points at the level of 80 keV (332); image quality had 302 at this level and vascular contrast received 197. The level of 90 keV received a total of 330 for noise, 252 for overall image quality, and 157 for vascular contrast. From there, the summed up ratings decrease further.

## 4. Discussion

The aim of this study was to examine virtual monoenergetic reconstructions at different keV levels of pv phase PCD-CT spectral datasets to evaluate the vascular contrast in intra-abdominal arteries compared with pc arterial phase datasets. We found 40 keV to have higher vessel contrast than the pc arterial phase, concomitant with significantly elevated noise and reduced CNR and SNR. Attenuation at VMI 40 keV was 18% higher than in pc images of the arterial phase with a statistical significance this is in line with the subjective rating of vascular contrast. The best subjective results for noise were found at 80 keV and for overall quality the best results were found at 70 keV (image noise showed a steady decrease even above the levels of 70 keV, asymptotically reaching around 5 at 140 keV and above; also, image noise was lower than in pc arterial images at the levels of 70 keV and higher). Above the energy threshold of 100 keV, attenuation in pv VMI shows values less than 100 HU. A possible reason for the decrease of subjective image quality with higher energy thresholds might be the lack of vascular contrast, therefore creating an almost non-enhanced scan impression.

We showed that the pv energy threshold of 40 keV had significantly higher attenuation than the pc images of the arterial phase. This is in accordance with the research of Lennartz et al. [29], which was performed on a dual-layer spectral detector CT scanner. They showed higher attenuation in VMI without statistical significance; the increase in attenuation was comparable to our results but we saw higher absolute values for 40 keV in PCD-CT compared to dual layer spectral CT (e.g., aorta PCD-CT 470 ± 94 HU vs. dual layer spectral detector 369 ± 90 HU).

The value of pv VMI at 40 keV noise in our study was higher, and the CNR and SNR pv VMI at 40 keV showed lower values compared to the arterial pc images, which is different from the results of Lennartz et al. and Patel et al. [29,30]. The research of Patel et al. showed a non-inferiority for attenuation in VMI compared with true arterial images (also regarding SNR and CNR) for all intra-abdominal vessels on a dual-layer spectral CT scanner [30]. Their CNR and SNR reached a maximum at the VMI level of 40 keV (as in our research) but they used the standard deviation of fat, not air, to calculate the CNR.

Reconstructing images at VMI 40 keV might be an option for optimizing vascular contrast in the case of a missed contrast medium bolus (e.g., in a runoff angiography, as the missing surrounding soft tissue might allow better CNRs and SNRs than in intra-abdominal vessels).

Sartoretti et al. [31] showed on a PCD-CT that there is a possibility of a significant improvement of CNR, SNR, and subjective image quality by using higher levels of iterative reconstruction at the energy level of 60 keV and pc images. Racine et al. [32] also showed better noise at higher levels. Noise was already reduced by the use of Mono+ mode as previously described for dual energy CT scanners, which explains the difference compared to previous VMI research [33,34].

Our overall pv attenuation values demonstrated a decline below 230 HU, beginning at the 60 keV level (212 ± 42 HU) with a further reduction of 80% in vessel attenuation to 190 keV (43 ± 10 HU). Previously, 230 HU was described as the cut-off for sufficient arterial contrast [35], and other studies suggest even higher attenuation as being sufficient for imaging of the thoracic aorta (250–300 HU)) [36,37,38].

Higher energy levels showed effects on reducing metal artifacts in patients, e.g., with hip replacements, metal artifact reconstructions could add to this effect [39,40,41,42]. But as Neuhaus et al. showed, 140 keV is an optimal level for metal artifact reduction by VMI [40]. Reconstructing images at this level in PV does not appear to be a viable alternative for reducing metal artefacts with the aim of assessing arteries, as we only saw an average of 55 HU at this energy threshold.

Our findings indicate comparatively lower values for both CNR and SNR in contrast to the study conducted by Graafen et al., which examined true arterial phase PCD-CT versus EID-CT for arterial abdominal imaging. Specifically, for CNR at 40 keV, our results were 8.71 ± 3.53, whereas Graafen et al. reported 23.9 ± 6.7. This result can be attributed to the true arterial phase and the consequent higher iodine levels, as analyzed by Graafen et al., compared to the pv phase in our study [19,33].

Our study has some limitations. First, the number of included patients could be increased (*n* = 40); still, the number we used is big enough to show reliable results. Future studies could focus on larger patient cohorts.

Additionally, we did not focus on the effect of iterative reconstruction, which can have a significant effect on objective and subjective image quality, including SNR and CNR, as Sartoretti et al. have shown [31].

## 5. Conclusions

Our results suggest that VMI of a pv phase at 40 keV could be a feasible alternative for the assessment of arterial intra-abdominal vessels with an 18% higher contrast than that of true arterial pc images at the cost of elevated noise and reduced CNR and SNR. This could be a chance to reduce patient dose by preventing re-examinations (e.g., vascular pathologies, found in a pv phase) or maybe even waiving a true arterial phase in selected cases.

VMI showed the best subjective parameters at 70 keV with a better CNR and SNR than at 40 keV; 70 keV also marks the break-even point regarding noise in pc arterial and VMI pv images.

## Figures and Tables

**Table 1:** Population data of the scans (CTDI = computed tomography dose index, DLP = dose length product).

Parameter	Overall Data	Male	Female
age (years)	68 ± 13	68 ± 12	69 ± 16
calculated diameter (cm)	30.71 ± 3.81	30.83 ± 3.19	30.49 ± 4.9
CTDI (mGy)	6.17 ± 1.99	6.02 ± 1.74	6.45 ± 2.44
DLP (mGy × cm)	292.35 ± 105.56	295.5 ± 105.06	286.5 ± 110.12

**Table 2:** Demonstration and comparison of the means and standard deviations (SD) of the measured objective image parameters of VMI pv phase on different energy levels and pc arterial values (pv = portalvenous; pc = polychromatic; HU = Hounsfield Units; CNR = contrast-to-noise-ratio; SNR = signal-to-noise-ratio).

	keV	pv Mean and SD	pc Arterial Mean and SD
HU	40 and pc	440 ± 97	360 ± 97
	50	298 ± 63	
	60	212 ± 42	
	70	159 ± 29	
	80	124 ± 22	
	90	101 ± 17	
	100	85 ± 15	
	110	74 ± 13	
	120	66 ± 12	
	130	59 ± 11	
	140	55 ± 11	
	150	51 ± 11	
	160	48 ± 10	
	170	46 ± 10	
	180	44 ± 10	
	190	43 ± 10	
noise	40 and pc	63.27 ± 16.05	19.95 ± 8.58
	50	41.31 ± 10.25	
	60	27.17 ± 6.78	
	70	19.22 ± 4.81	
	80	13.63 ± 3.87	
	90	10.19 ± 3.40	
	100	8.08 ± 3.21	
	110	6.80 ± 3.15	
	120	6.05 ± 3.15	
	130	5.62 ± 3.16	
	140	5.40 ± 3.18	
	150	5.30 ± 3.19	
	160	5.26 ± 3.20	
	170	5.27 ± 3.20	
	180	5.32 ± 3.20	
	190	5.37 ± 3.21	
CNR	40 and pc	8.71 ± 3.53	25.10 ± 9.38
	50	8.49 ± 3.46	
	60	8.63 ± 3.58	
	70	8.27 ± 3.44	
	80	7.75 ± 3.37	
	90	7.21 ± 3.51	
	100	6.52 ± 3.74	
	110	5.64 ± 3.98	
	120	4.55 ± 4.18	
	130	3.35 ± 4.34	
	140	2.14 ± 4.47	
	150	1.04 ± 4.57	
	160	0.10 ± 4.62	
	170	−0.66 ± 4.60	
	180	−1.26 ± 4.53	
	190	−1.73 ± 4.49	
SNR	40 and pc	7.42 ± 2.73	20.10 ± 8.30
	50	7.68 ± 2.72	
	60	8.28 ± 2.73	
	70	8.73 ± 2.71	
	80	9.73 ± 3.15	
	90	10.79 ± 3.70	
	100	11.77 ± 4.39	
	110	12.57 ± 5.24	
	120	13.09 ± 5.24	
	130	13.20 ± 6.88	
	140	13.06 ± 7.50	
	150	12.73 ± 7.98	
	160	12.25 ± 8.07	
	170	11.70 ± 7.84	
	180	11.08 ± 7.45	
	190	10.59 ± 7.13	

**Table 3:** Comparing the attenuation of VMI pv 40 keV with pc images grouped by vessel with the corresponding paired samples *t*-test *p*-value.

Vessel		Mean Attenuation ± Standard Deviation	*p*-Value
aorta	HU.art pc	356 ± 96	<0.001
	HU.pv.40	470 ± 96	
coeliac trunk	HU.art pc	362 ± 100	<0.001
	HU.pv.40	440 ± 94	
superior mesenteric artery	HU.art pc	368 ± 103	<0.001
	HU.pv.40	436 ± 87	
left iliac artery	HU.art pc	368 ± 96	<0.001
	HU.pv.40	466 ± 96	
right iliac artery	HU.art pc	365 ± 96	<0.001
	HU.pv.40	465 ± 96	
right renal artery	HU.art pc	353 ± 97	<0.001
	HU.pv.40	404 ± 91	
left renal artery	HU.art pc	345 ± 92	<0.001
	HU.pv.40	397 ± 95	

## Data Availability

Detailed data available on request. The data are not publicly available due to ethical restrictions.
